# Macrophage Activation Redirects *Yersinia*-Infected Host Cell Death from Apoptosis to Caspase-1-Dependent Pyroptosis

**DOI:** 10.1371/journal.ppat.0030161

**Published:** 2007-11-02

**Authors:** Tessa Bergsbaken, Brad T Cookson

**Affiliations:** 1 Department of Microbiology, University of Washington, Seattle, Washington, United States of America; 2 Department of Laboratory Medicine, University of Washington, Seattle, Washington, United States of America; Tufts University School of Medicine, United States of America

## Abstract

Infection of macrophages by *Yersinia* species results in YopJ-dependent apoptosis, and naïve macrophages are highly susceptible to this form of cell death. Previous studies have demonstrated that macrophages activated with lipopolysaccharide (LPS) prior to infection are resistant to YopJ-dependent cell death; we found this simultaneously renders macrophages susceptible to killing by YopJ^−^ Yersinia pseudotuberculosis (*Yptb*). YopJ^−^ *Yptb*-induced macrophage death was dependent on caspase-1 activation, resulting in rapid permeability to small molecules, followed by membrane breakdown and DNA damage, and accompanied by cleavage and release of proinflammatory interleukin-18. Induction of caspase-1-dependent death, or pyroptosis, required the bacterial type III translocon but none of its known translocated proteins. Wild-type *Yptb* infection also triggered pyroptosis: YopJ-dependent activation of proapoptotic caspase-3 was significantly delayed in activated macrophages and resulted in caspase-1-dependent pyroptosis. The transition to susceptibility was not limited to LPS activation; it was also seen in macrophages activated with other Toll-like receptor (TLR) ligands and intact nonviable bacteria. *Yptb* infection triggered macrophage activation and activation of caspase-1 in vivo. Y. pestis infection of activated macrophages also stimulated caspase-1 activation. These results indicate that host signaling triggered by TLR and other activating ligands during the course of *Yersinia* infection redirects both the mechanism of host cell death and the downstream consequences of death by shifting from noninflammatory apoptosis to inflammatory pyroptosis.

## Author Summary

Pathogenic *Yersinia* are bacteria capable of interacting with host immune cells and inhibiting their function. Macrophages are potent antimicrobial immune cells that eliminate invading microbes, and represent a major target for *Yersinia* during infection. *Yersinia* triggers death of resting macrophages by apoptosis, a process thought to be advantageous for *Yersinia* growth during early stages of infection because important host cells are eliminated without perturbing the surrounding tissue. However, activated macrophages with enhanced antimicrobial activity play a crucial role in controlling *Yersinia* infection. To elucidate the mechanisms involved in successful defense against infection, the authors investigated the response of activated macrophages to *Yersinia*, which revealed induction of a proinflammatory cell death pathway termed pyroptosis. Unlike apoptosis, pyroptosis unleashes inflammatory mediators capable of enhancing immune responses and clearing bacteria*.* Macrophage activation and pyroptosis was observed in infected host tissue. Thus, regulating the mechanism of cell death is important for effective responses to *Yersinia* infection: activated macrophages resisting apoptosis are redirected to utilize pyroptosis, an inflammatory process facilitating host resistance.

## Introduction

The genus *Yersinia* includes three species pathogenic for humans: Yersinia pestis, the causative agent of plague, and Y. entercolitica and Y. pseudotuberculosis, which cause gastroenteritis and lymphadenitis and occasionally systemic infection. All pathogenic *Yersinia* species harbor a 70 kb virulence plasmid, which encodes a type III secretion system (T3SS) and the effector proteins translocated by this system [1]. The structural components of the T3SS include the needle complex and the secreted proteins YopB and YopD, which form a conduit through the host cell membrane to allow entry of bacterial effector proteins directly into the host cytosol. Once the effector proteins (Yops E, H, O, M, and J) reach the cytosol, they function primarily to inhibit phagocytosis and suppress the host inflammatory response triggered upon bacterial interaction [2]. In addition, all three pathogenic *Yersinia* species are able to induce cell death in naïve macrophages, and this requires the translocated effector YopJ [3–5].

Two signals are required for maximal induction of cell death in *Yersinia-*infected naïve macrophages, YopJ and signaling through host Toll-like receptor 4 (TLR4) [6,7]. Upon contact with a macrophage, *Yersinia* lipopolysaccharide (LPS) recognized by host TLR4 simultaneously initiates apoptotic signaling through the adapter protein TRIF [8,9] as well as mitogen-activated protein kinase (MAPK)- and nuclear factor kappa B (NF-κB)-dependent up-regulation of inflammatory cytokine production and cell survival genes [10–12]. However, YopJ inhibits the activation of NF-κB and MAPKs [13–15], allowing apoptotic signaling to predominate. TRIF-dependent signaling leads to cleavage of the apoptotic initiator caspase-8 [9] and release of cytochrome c from the mitochondria [16]. This leads to activation of downstream executioner caspases-9, −7, and −3 and apoptosis of *Yersinia*-infected naïve macrophages [16]. Although inducing cell death via apoptosis could potentially suppress inflammation and eliminate macrophages, a host cell type hypothesized to play an important role in combating *Yersinia* infection [5,17], the relative importance of these YopJ-dependent processes during *Yersinia* infection is somewhat controversial. Some groups report no change in virulence of YopJ mutant bacteria [18–20] and others observe varying degrees of attenuation [21,22].

In vivo, *Yersinia* delays inflammation in a T3SS-dependent manner, allowing the bacteria to proliferate [23]. However, the pathology resulting from infection is strikingly biphasic: as infection progresses, *Yersinia* no longer controls the inflammatory host response resulting in the influx of neutrophils and macrophages, increased inflammatory cytokine production, and tissue necrosis [24–28]. The presence of both host inflammatory mediators and/or bacterial products during infection could result in macrophage activation [29], which is thought to play a critical role in the resolution of *Yersinia* infection [5,17]. Pretreatment of mice with the macrophage-activating cytokines tumor necrosis factor alpha and interferon gamma confers protection to lethal challenge [23], and dysregulation of the normal LPS modifications made by *Yersinia* increases their stimulation of macrophages and allows the host to control infection [30]. This suggests that a greater understanding of the differing responses of naïve and activated macrophages to *Yersinia* infection will provide insight into the immunopathogenesis involved in establishing an ongoing infection, as well as generating protective host immune responses. Activation of macrophages alters their adhesion, migration, and cytokine production, and increases antigen endocytosis, antigen presentation, and activation of effector functions [29]. Importantly, macrophage activation alters the cellular response to death inducing stimuli [10,12,31,32]. Previous studies indicate the toxic effects of YopJ are altered in activated macrophages: LPS pretreated macrophages activate NF-κB, a process inhibited by YopJ in naïve macrophages, and YopJ-dependent apoptosis is correspondingly reduced [33].

In this study we examined the response of activated macrophages to Y. pseudotuberculosis (*Yptb*) infection in vitro and in vivo*.* TLR-mediated suppression of apoptosis leads to *Yptb-*induced macrophage cell death being redirected to a caspase-1-dependent inflammatory pathway called pyroptosis [34], and this process depends upon an intact *Yptb* T3SS. We also observed the characteristics of this mechanistic shift in host cell execution, macrophage activation and activation of caspase-1, occurring during *Yptb* infection in vivo*.* Together our observations suggest modulation of host cell death pathways is an important response to infection.

## Results

### Activation of Macrophages Increases Susceptibility to Cell Death Induced by YopJ^−^ *Yptb*

**Figure 1:**
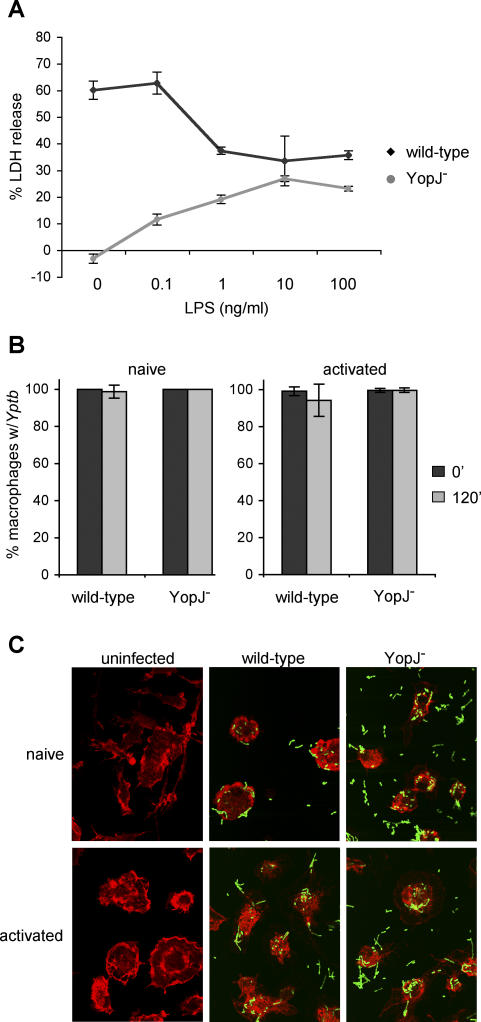
Activation of Macrophages Increases Susceptibility to Cell Death Induced by Infection with YopJ^−^ *Yptb* BMDMs were treated with varying concentrations of LPS for 18 h prior to infection with wild-type or YopJ^−^ *Yptb*. (A) Host cell lysis was assessed by measuring release of cytosolic LDH into the supernatant at 3.5 h postinfection. Data shown are means and SDs calculated from three replicates and are representative of three experiments. (B and C) Naïve macrophages and macrophages activated with 100 ng/ml LPS for 18 h were infected with GFP-expressing wild-type or YopJ^−^ *Yptb.* The uniformity of host cell infection was assessed by confocal microscopy. The percentage of macrophages with associated *Yptb* immediately after (black bars) and 120 min (gray bars) postinfection was determined (B). Means and SDs were calculated from more than five fields with a minimum of 150 cells for each condition. GFP-expressing *Yptb* are shown interacting with host cells at 120 min postinfection (C). Host cells were visualized by staining actin with Texas Red-phalloidin; representative images are shown.

Infection of macrophages with *Yptb* results in the induction of apoptosis that is dependent on the effector YopJ [3,4,35]. Treatment of macrophages with LPS prior to infection with *Yersinia* has been shown to decrease YopJ-dependent apoptosis [33]. We confirmed that LPS pretreatment of macrophages (macrophage activation) [29] prior to infection with wild-type *Yptb* reduced macrophage cell death by approximately 50% as measured by release of cytosolic lactate dehydrogenase (LDH) (Figure 1A). In addition, macrophage activation increased LDH release from background levels to 30% during infection with a *yopJ* mutant. Both phenotypes were observed at LPS concentrations as low as 1 ng/ml (Figure 1A).

Altered sensitivity of activated macrophages to cell death induced by *Yptb* infection may result from a change in *Yptb*:macrophage interactions; therefore, the ability of *Yptb* to interact with naïve and activated macrophages was compared. Naïve and LPS-activated macrophages were infected with green fluorescent protein (GFP)-expressing wild-type or YopJ^−^ *Yptb* and examined by microscopy. For all conditions, greater than 98% of macrophages had one or more associated bacteria immediately after infection (Figure 1B). At 2 h postinfection, over 94% of macrophages were associated with multiple bacteria (Figure 1B and 1C). Additionally, translocation of effector proteins into the cytosol of naïve and activated macrophages was measured during infection with *Yptb* expressing YopE fused to the Bordetella pertussis calmodulin-dependent adenylate cyclase (Cya), resulting in accumulation of cAMP when YopE-Cya reaches the host cell cytosol [36]. Activation of macrophages did not reduce the level of effector translocation (unpublished data). These results demonstrated that activation of macrophages did not affect the ability of *Yptb* to associate with macrophages or translocate effectors, but appeared to alter the cellular response to infection.

### *Yptb*-Induced Cell Death in Activated Macrophages Results in Rapid Membrane Permeability Preceding DNA Damage

**Figure 2:**
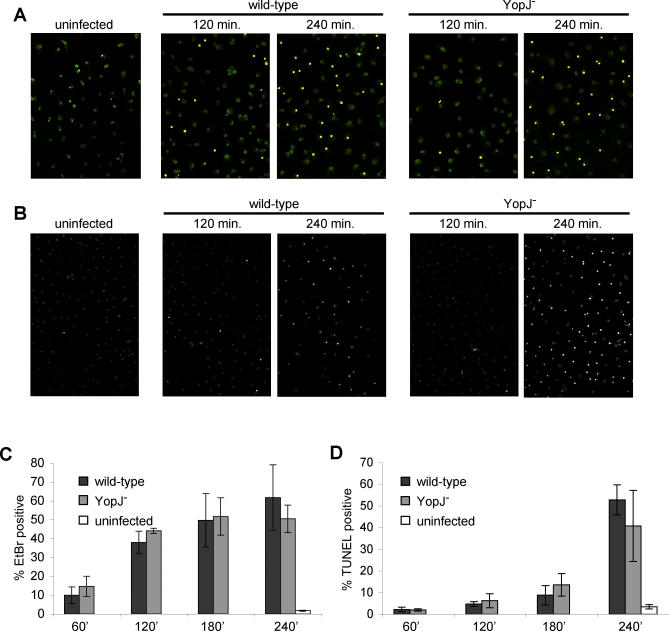
Wild-Type and YopJ^−^ *Yptb* Infection of Activated Macrophages Stimulates Membrane Permeabilization Followed by DNA Damage The kinetics of cell death were examined in LPS-activated BMDMs infected with wild-type and YopJ^−^ *Yptb*. (A) Macrophages labeled with SYTO10 (green) were stained with membrane-impermeant EtBr (MW = 394 Da, red) and examined by confocal microscopy to assess increases in membrane permeability (EtBr-positive/SYTO10-labeled, yellow). Representative images are shown. (B) DNA damage was also assessed by TUNEL and confocal microscopy in the same experiment. Representative images are shown. (C) The percentage of EtBr-positive/SYTO10-labeled cells was determined; data shown are means and SDs from four or more fields with a minimum of 350 cells total for each time point. Results shown are representative of two experiments. (D) The percentage of TUNEL-positive cells was determined; data shown are means and SDs from four or more fields with a minimum of 450 cells total for each time point. Results shown are representative of two experiments.

The similar levels of LDH release observed with wild-type and YopJ^−^ *Yptb* infection of activated macrophages (Figure 1A) led us to hypothesize that the same process occurred during infection with both strains. We therefore assessed the kinetics of two nonspecific markers of cell death, membrane damage and DNA damage [37], during infection of activated macrophages with wild-type and YopJ^−^ *Yptb.* Using uptake of a small membrane impermeant dye, ethidium bromide (EtBr, MW = 394 Da), allowed us to quantitatively examine membrane damage in individual cells during infection. EtBr uptake in uninfected macrophages was less than 2% (Figure 2A and 2C). Infection with wild-type or YopJ^−^ *Yptb* resulted in very similar kinetics of EtBr uptake: 10%–15% cells were EtBr+ cells at 60 min postinfection, and this increased to 40%–45% EtBr+ cells by 120 min, and did not increase in the next 120 min of infection (Figure 2C). We used terminal deoxynucleotidyl transferase-nick end labeling (TUNEL) to examine DNA damage, and again, the kinetics were nearly identical during infection with both strains (Figure 2B and 2D). The percent of TUNEL-positive cells remained low until 180 min after infection; 15% of infected macrophages were TUNEL-positive at this time point. This increased to greater than 40% by 240 min, compared to less than 2% in uninfected macrophages (Figure 2B and 2D).

Similar kinetics of wild-type and YopJ^−^ *Yptb* stimulated membrane and DNA damage during infection of activated macrophages is consistent with our hypothesis that both strains activate the same process. Importantly, membrane damage significantly preceded DNA damage (Figure 2). This observation is in contradistinction to YopJ-dependent apoptosis induced during wild-type *Yptb* infection of naïve macrophages, which has been previously described [3,4,11,16,35]. DNA damage began before membrane damage during wild-type *Yptb* induction of apoptosis in naïve macrophages (Figure S1A–S1D), and as shown in Figure 1A, YopJ^−^ *Yptb* was unable to kill naïve macrophages. As expected, induction of apoptosis by wild-type *Yptb* activates apoptotic caspases-3 and −8 [9,16], a caspase-3 inhibitor correspondingly inhibits DNA damage, and also as shown previously [6,7], TLR4 signaling facilitates apoptosis during wild-type *Yptb* infection of naïve macrophages (Figure S1E–S1G). Finally, infection of naïve macrophages with wild-type *Yptb* results in the typical nuclear condensation characteristic of apoptosis (Figure S2A) [3,4,35], in contrast to the diffuse distribution of TUNEL-positive DNA observed in activated macrophages infected with wild-type or YopJ^−^ *Yptb* (Figure S2B). Thus, the features of wild-type and YopJ^−^ *Yptb*-induced cell death in activated macrophages, which include membrane permeability preceding DNA damage with morphological features excluding apoptosis [38], suggest an alternative mechanism of cell death.

### YopJ^−^ *Yptb*-Induced Cell Death Requires Caspase-1

**Figure 3:**
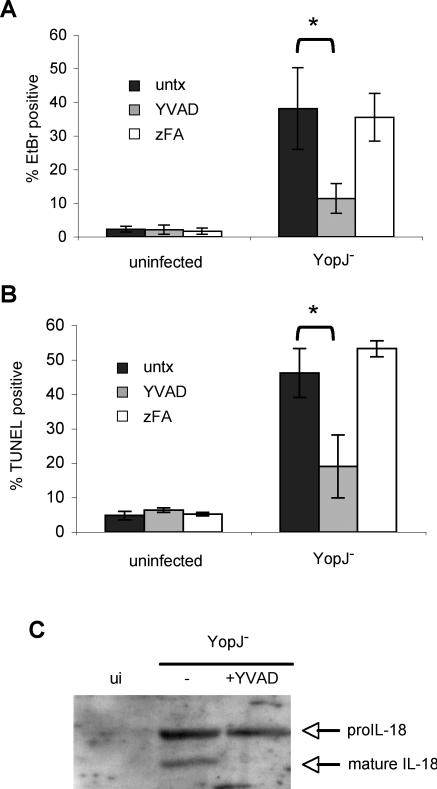
YopJ^−^ *Yptb-*Induced Membrane Permeability and DNA Damage Are Caspase-1-Dependent and Accompanied by Inflammatory Cytokine Processing LPS-activated BMDMs were treated with caspase-1 inhibitor (YVAD) or negative control (zFA) peptide (200 μM) during infection with YopJ^−^ *Yptb*. Membrane permeability was examined by SYTO10/EtBr staining (see Figure 2A legend) and confocal microscopy at 120 min postinfection (A). DNA damage was assessed using TUNEL and confocal microscopy at 240 min postinfection (B). The percentage of EtBr/TUNEL-positive cells was determined from four or more fields with a minimum of 1,000 cells total for each condition. Representative of two experiments. * *p* < 0.0005. (C) Western blot analysis of mature IL-18 released into the supernatant by activated macrophages at 90 min postinfection with YopJ^−^ *Yptb* confirms caspase-1 activation and cytokine processing. Representative of two experiments. ui, uninfected.

LPS activation renders macrophages susceptible to cell death induced by ATP treatment [32,39] or Francisella tularensis infection [40], and both processes involve caspase-1. The features of YopJ^−^ *Yptb*-induced cell death, rapid membrane permeability preceding DNA damage, and the lack of nuclear condensation, also suggest caspase-1-dependent cell death or pyroptosis [38,41]. We therefore hypothesized that YopJ^−^ *Yptb-*induced cell death was dependent on caspase-1. EtBr uptake by YopJ^−^ *Yptb* infected macrophages was reduced by the specific caspase-1 inhibitor YVAD [42], but not by the negative control peptide zFA (Figure 3A), indicating caspase-1 is required for increased membrane permeability during infection. Additionally, the downstream events of membrane breakdown and release of LDH were inhibited by YVAD (unpublished data). DNA damage was caspase-1-dependent; the percentage of TUNEL positive cells was reduced in the presence of YVAD and unchanged by zFA (Figure 3B). Features of wild-type *Yptb-*induced apoptosis in naïve macrophages were not inhibited by YVAD (unpublished data), indicating YVAD was not nonspecifically inhibiting *Yptb* or apoptotic caspases. Finally, we examined supernatants collected from activated macrophages infected with YopJ^−^ *Yptb* for the presence of the inflammatory cytokine interleukin (IL)-18, which is specifically cleaved and activated by caspase-1 [43]. Cleaved IL-18 was present in supernatants from macrophages infected with YopJ^−^ *Yptb*, but not uninfected macrophages, and IL-18 processing was blocked by YVAD (Figure 3C). This demonstrated that the features of YopJ^−^ *Yptb* induced cell death are caspase-1-dependent and accompanied by cleavage and release of the caspase-1 substrate IL-18.

### Induction of Pyroptosis Requires the Bacterial Type III Translocon, but None of the Known *Yptb* Effectors

**Figure 4:**
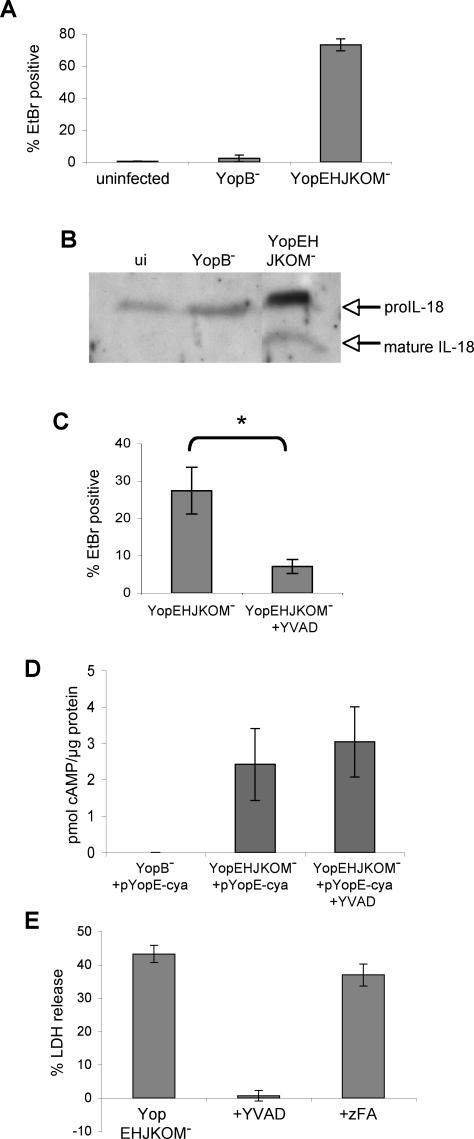
*Yptb-*Induced Pyroptosis Is T3SS-Dependent LPS-activated BMDMs were infected with YopEHJKOM^−^ (T3SS^+^, type III effector^−^) or YopB^−^ (T3SS^−^) *Yptb.* (A) Membrane permeability was examined at 90 min postinfection by EtBr/SYTO10 staining (See Figure 2A legend) and confocal microscopy. Data shown are from four or more fields with a minimum of 1,000 cells total for each condition. Representative of two experiments. (B) Western blot analysis of mature IL-18 released into the supernatant by infected macrophages at 90 min postinfection confirms caspase-1 activation. Representative of two experiments. ui, uninfected. (C) Membrane permeability was examined in infected macrophages treated with YVAD by SYTO10/EtBr staining and confocal microscopy at 45 min postinfection. Data shown are from four or more fields with a minimum of 1,000 cells for each condition. Representative of three experiments. * *p* < 0.0001. (D) Translocation of a YopE-adenylate cyclase fusion protein into the cytoplasm of activated macrophages was assessed by quantifying cAMP levels at 60 min postinfection, and demonstrated equivalent levels of effector translocation by YopEHJKOM^−^ *Yptb* in the presence of YVAD. Data shown are means and SDs calculated from three replicates. (E) Membrane breakdown and LDH release from macrophages treated with YVAD or negative control peptide zFA 120 min postinfection with YopEHJKOM^−^ *Yptb*. Data shown are means and SDs calculated from three replicates and are representative of three experiments.

Pyroptosis induced by several other bacteria requires a functional T3SS [38,44–47]. YopB and YopD are structural components of the *Yersinia* type III translocon, and both are required for translocation of effector proteins into host cells [2]. To examine the requirement for the T3SS in *Yptb-*induced pyroptosis, activated macrophages were infected with YopB^−^ *Yptb*; this mutant was unable to alter host membrane permeability (Figure 4A). In addition to YopJ, *Yersinia* translocates several other effectors into the macrophage cytosol (Yops E, H, O, and M) [2], and we examined the role of these effector proteins in inducing pyroptosis. Infection of activated macrophages with YopEHJKOM^−^ *Yptb*, a mutant lacking all of the known translocated effectors but competent for type III translocation (YopBD+), increased macrophage membrane permeability and uptake of EtBr (Figure 4A). Furthermore, infection by YopEHJKOM^−^ *Yptb*, but not YopB^−^ *Yptb*, resulted in release of cleaved IL-18 (Figure 4B). Infection with *Yersinia* mutants lacking multiple effectors, but competent for type III translocation (YopBD+), results in pore formation in the host cell membrane and uptake of small molecules similar to EtBr in size [48,49]. This pore was thought to be the type III translocon composed of YopB and YopD. However, caspase-1 activation can lead to formation of membrane pores [41], and we hypothesized pore formation by YopEHJKOM^−^ *Yptb* was instead a host-mediated process dependent upon caspase-1. Consistent with this idea, YVAD inhibited EtBr uptake by activated macrophages infected with YopEHJKOM^−^ *Yptb* (Figure 4C). Importantly, we demonstrated the YopB/D translocation pore is formed and functional in the presence and absence of YVAD; a YopE-Cya fusion protein is translocated equally in both conditions (Figure 4D). Additionally, membrane breakdown and LDH release was completely inhibited by YVAD, but not zFA (Figure 4E). These data indicate pyroptosis induced by *Yptb* requires the T3SS, but none of its known translocated effector proteins, and host cell membrane damage and EtBr uptake are caspase-1-mediated processes stimulated by *Yptb* infection.

### Macrophage Activation Antagonizes Wild-Type *Yptb*-Induced Apoptosis and Stimulates Pyroptosis

**Figure 5:**
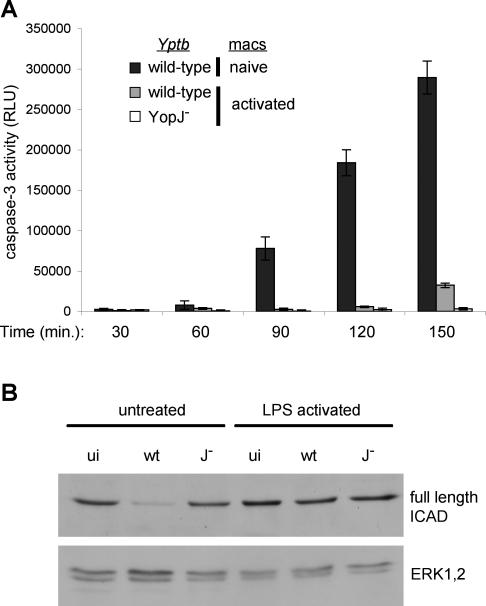
Macrophage Activation Antagonizes Caspase-3 Activity and Apoptosis during Infection with Wild-Type *Yptb* Untreated and LPS-activated BMDMs were infected with wild-type or YopJ^−^ *Yptb*. (A) Kinetics of caspase-3 activation in infected macrophages. Data shown are means and SDs calculated from three replicates and presented as relative light units (RLU) in cell lysates from infected samples minus uninfected controls. Representative of three experiments. At 150 min postinfection, caspase-3 activity in naïve macrophages undergoing YopJ-dependent apoptosis during infection with wild-type *Yptb* (289,340 ± 20,466 RLU) is greatly reduced in activated macrophages infected with wild-type *Yptb* (32,315 ± 2,881 RLU). Activated macrophages infected with YopJ− *Yptb* fail to activate caspase-3 (3,231 ± 1,531 RLU). (B) Cleavage of the caspase-3 substrate ICAD was examined in uninfected macrophages (ui) and at 120 min postinfection with wild-type (wt) or YopJ^−^ (J^−^) *Yptb* by Western blot and confirmed the absence of caspase-3 activity in activated macrophages. ERK1/2 was used as a loading control.

We observed pyroptosis in activated macrophages infected with *Yptb* that lack YopJ but contain a functional T3SS. During infection of naïve macrophages with wild-type *Yptb*, YopJ and TLR4 signaling are required for maximal activation of caspase-3 and apoptosis [6,7,9]. However, macrophage activation can dampen future TLR4-mediated signaling events [50,51], and result in synthesis of gene products that inhibit the activation/activity of apoptotic caspases, including caspase-3 [10–12]. We therefore hypothesized that macrophage activation would decrease YopJ-dependent caspase-3 activation and apoptosis during subsequent infection with wild-type *Yptb*, and simultaneously stimulate pyroptosis. As expected, wild-type *Yptb* infection of naïve macrophages resulted in rapid caspase-3 activation (Figure 5A) and cleavage of the caspase-3 substrate inhibitor of caspase-activated DNase (ICAD) [52] (Figure 5B, left). Infection with YopJ^−^ *Yptb* did not result in caspase-3 activity (Figure 5A) or degradation of ICAD (Figure 5B), regardless of the activation state of the macrophages. However, in activated macrophages infected with wild-type *Yptb*, caspase-3 activity was undetectable until 150 min postinfection (Figure 5A) and no ICAD degradation was detected (Figure 5B, right), which together confirm the lack of caspase-3 activity in these infected cells (Figure 5A).

**Figure 6:**
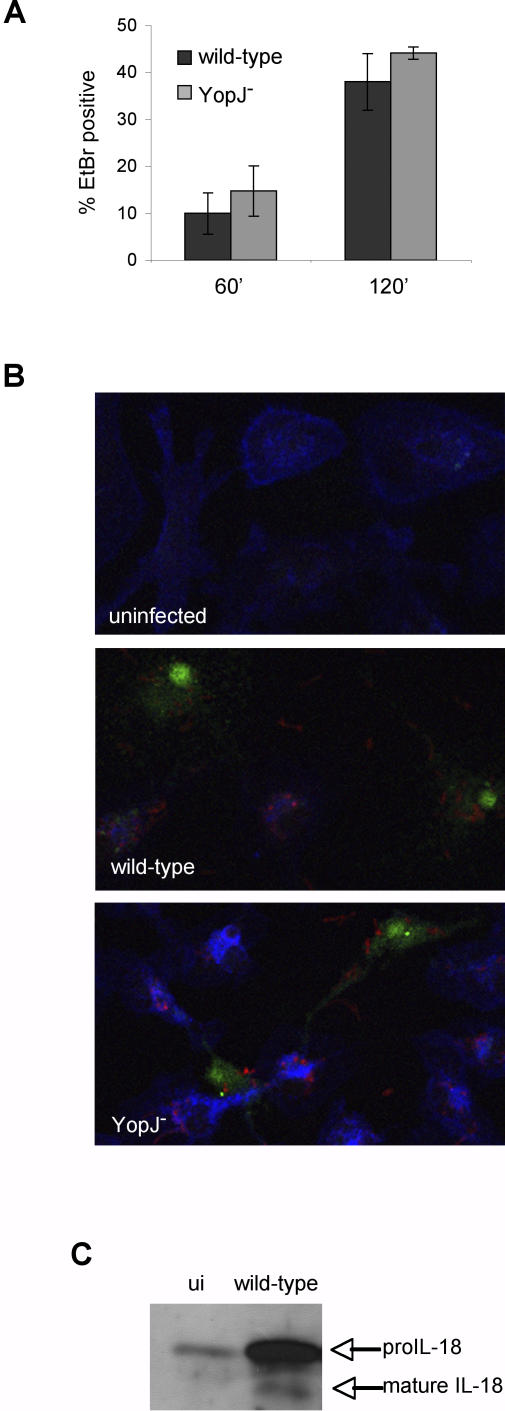
Wild-Type *Yptb* Infection Induces Pyroptosis of Activated Macrophages LPS-activated BMDMs were infected with wild-type or YopJ^−^ *Yptb*. (A) The kinetics of membrane permeability were examined by EtBr/SYTO10 staining (see Figure 2B legend) and confocal microscopy. Data shown are from four or more fields with a minimum of 350 cells for each time point. Representative of three experiments. (B) Macrophages were stained with FAM-YVAD (green) to identify cells with active caspase-1, Alexa633-phalloidin to visualize actin (blue), and anti-*Yersinia* antibodies (red) 90 min postinfection and examined by confocal microscopy. Representative images are shown. (C) Western blot analysis of mature IL-18 released into the supernatant at 90 min postinfection confirmed caspase-1 activation by wild-type *Yptb* infection. Representative of two experiments. ui, uninfected.

An early feature of pyroptosis is permeability to EtBr (Figure 2A and 2B), and activated macrophages infected with wild-type or YopJ^−^ *Yptb* became permeable to EtBr with identical kinetics (Figure 6A). Importantly, wild-type *Yptb* infection of activated macrophages resulted in EtBr uptake prior to any detectable increase in caspase-3 activity (Figure 5A), unlike infection of naïve macrophages, where caspase-3 activity precedes EtBr uptake (unpublished data). Activation of caspase-1 was examined by infecting macrophages in the presence of a fluorescently labeled inhibitor that irreversibly binds active caspase-1 (FAM-YVAD) [53]. Both wild-type and YopJ^−^ *Yptb* infection resulted in FAM-YVAD staining (Figure 6B), and wild-type *Yptb* infection of activated macrophages also induced cleavage and release of the caspase-1 substrate IL-18 into the supernatant (Figure 6C). These data confirmed that activation of macrophages prior to infection alters host cell responses to wild-type *Yptb*, suppressing YopJ-dependent apoptosis and simultaneously enhancing pyroptosis, resulting in caspase-1 activation, increased membrane permeability, and release of bioactive IL-18 prior to any detectable caspase-3 activation. Activation of macrophages in vitro alters susceptibility to cell death, and redirects infected macrophages to utilize the inflammatory pyroptosis pathway.

### Physiologically Relevant Stimuli Enhance Macrophage Susceptibility to *Yptb*-Induced Pyroptosis

**Figure 7:**
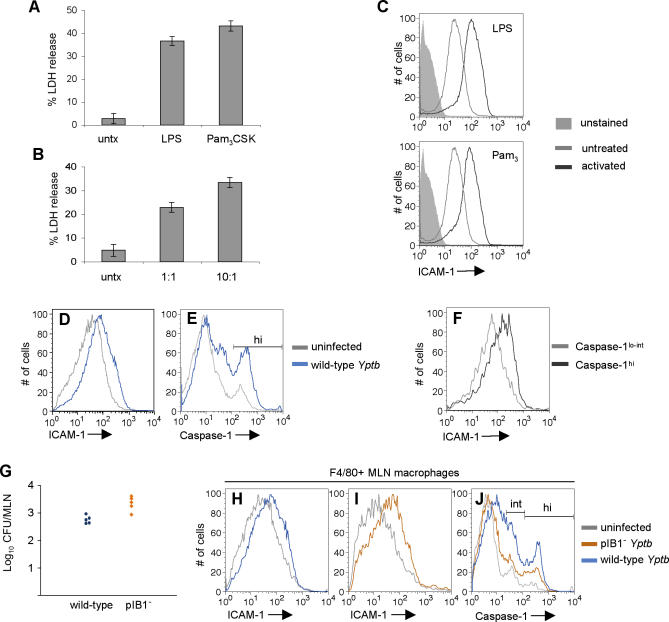
Macrophages Become Activated and Susceptible to Pyroptosis In Vivo during Murine Infection with *Yptb* (A and B) BMDMs were treated with 100 ng/ml LPS, 100 ng/ml Pam_3_CSK_4_ (A), or heat-killed *Yptb* at ratios of 10:1 or 1:1 *Yptb*:macrophage (B) for 18 h and infected with YopJ^−^ *Yptb*. LDH release was measured after 3.5 h. Data shown are from three replicates and representative of three experiments. (C) Macrophages were activated as in (A) and surface ICAM-1 expression was measured by flow cytometry. (D–F) At 4–6 d postinfection, activated splenic macrophages from wild-type *Yptb*-infected mice were identified with anti-F4/80, and ICAM-1 expression and caspase-1 activity (FAM-YVAD) were measured by flow cytometry. Representative histograms of macrophage ICAM-1 expression (D), caspase-1 activity (E), and ICAM-1 expression of the caspase-1^hi^ and caspase-1^lo-int^ (F) macrophage populations from (E). (G–J) Mice were infected with wild-type or pIB1^−^ *Yptb* and tissues were examined 5 d post-infection. CFU in the MLNs were quantified (G). Macrophages from the MLNs were identified with anti-F4/80, and ICAM-1 expression and caspase-1 activity were examined by flow cytometry. Representative histograms of MLN macrophage ICAM-1 expression from wild-type *Yptb*-infected (H) and pIB1^−^ *Yptb*-infected (I) mice; caspase-1 activity (J).

In addition to LPS, other TLR ligands are present during *Yptb* infection in vivo, and may activate macrophages and increase their sensitivity to pyroptosis. Activation of macrophages with a TLR2 ligand (Pam_3_CSK) prior to infection with YopJ^−^ *Yptb* increased pyroptosis to levels equivalent to LPS activation (Figure 7A). Pretreatment with whole heat-killed *Yptb*, which contain both TLR2 and TLR4 ligands [33], at ratios as low as one *Yptb* per macrophage also enhanced pyroptosis (Figure 7B). The TLR3 ligand poly(I:C) had a similar effect (unpublished data); although signaling through TLR3 may not be relevant in the context of *Yptb* infection, it supports the hypothesis that the redirection of macrophage death is a generalized host response to TLR stimulation.

### In Vivo Activation of Macrophages and Caspase-1 during *Yptb* Infection

We hypothesized that the abundance of activating ligands during *Yptb* infection would result in macrophage activation in vivo. Macrophages activated in vitro with LPS or Pam_3_CSK express increased levels of surface ICAM-1 (Figure 7C), which was used to monitor activation of F4/80+ macrophages and their susceptibility to pyroptosis in vivo. In mice infected orally with wild-type *Yptb*, splenic macrophages express increased surface ICAM-1 (Figure 7D), and this was observed in six of 14 infected mice (Figure S3). This was also observed in the mesenteric lymph nodes (MLNs); 16 of 18 mice with colonized MLNs contained macrophages with increased surface ICAM-1 expression (Figure S3).

The activation state of macrophages from wild-type *Yptb* infected mice suggested that these macrophages would be susceptible to pyroptosis; therefore, caspase-1 activation was examined by FAM-YVAD staining of splenocytes directly ex vivo. We observed an increase in the percentage of F4/80+ macrophages that were caspase-1^hi^ (Figures 7E and S4; Table S1) from 12.2% (± 1.86%) in uninfected mice to 29.0% (± 5.76%) in infected mice with activated macrophages (*p* = 0.0009). Additionally, macrophages from *Yptb-*infected mice that had no detectable increase in surface ICAM-1 expression had caspase-1 activity similar to macrophages from uninfected mice (Figure S4; Table S1). Macrophages with high levels of active caspase-1 (caspase-1^hi^, Figure 7E) expressed greater surface ICAM-1 when compared to macrophages with lower levels of active caspase-1 from the same infected tissue (Figure 7F). This confirms the activation of macrophages during wild-type *Yptb* infection and demonstrates that macrophage activation is necessary for increased activation of caspase-1, and correlates with our in vitro data demonstrating that wild-type *Yptb* infection of activated macrophages results in caspase-1-dependent pyroptosis.

To address the T3SS dependence of caspase-1 activation in vivo, mice were infected with *Yptb* lacking the T3SS-encoding pIB1 virulence plasmid. pIB1^−^ *Yptb* do not induce pyroptosis in vitro (unpublished data), but colonize the MLNs of infected mice as well as wild-type *Yptb* (Figure 7G) [28,54], and cause macrophage activation in vivo as measured by the increased expression of ICAM-1 (Figure 7H, wild-type; Figure 7I, pIB1^−^). However, the percentage of caspase-1^int+hi^ macrophages from pIB1^−^ *Yptb*-infected mice is significantly less than in wild-type *Yptb* infected mice (27.6% ± 4.57% versus 42.2% ± 2.18%, *p* < 0.003; Figure 7J), and macrophages from pIB1^−^ *Yptb-*infected mice had levels of active caspase-1 similar to uninfected mice (27.6% ± 4.57% versus 23.0% ± 8.49%, *p* = 0.32). These results confirm the activation of caspase-1 during *Yptb* infection in vivo, and the requirement for the T3SS-encoding virulence plasmid to activate caspase-1, thereby implicating the bacterial T3SS in this process in vivo*.*

### Y. pestis Induces Caspase-1 Activation

**Figure 8:**
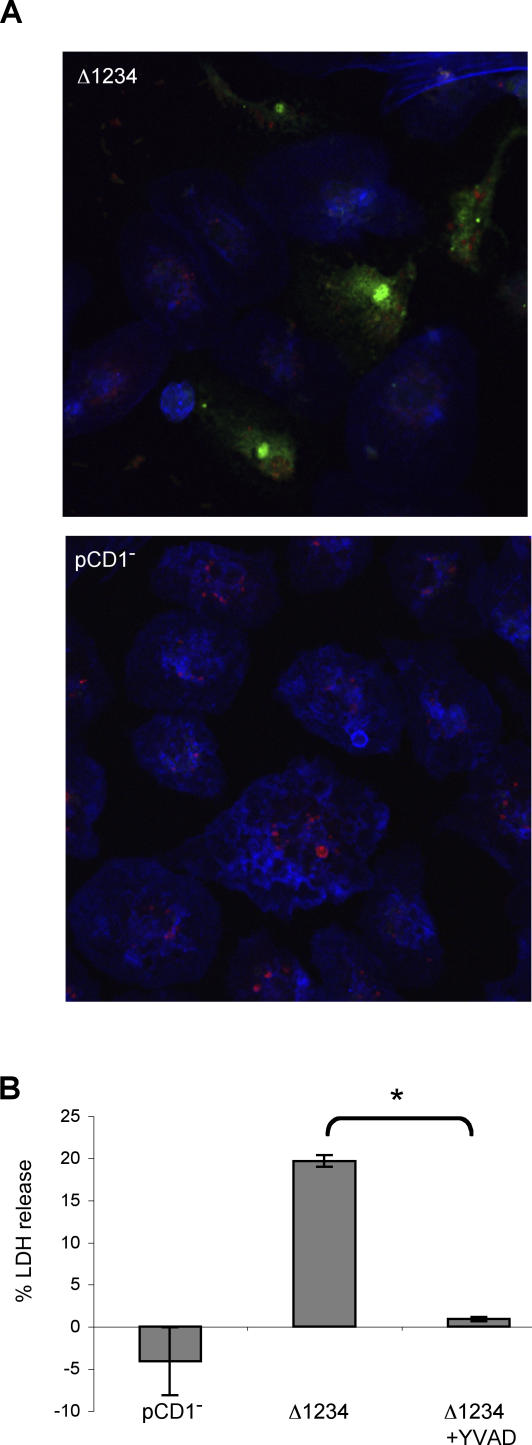
Y. pestis Infection Induces Pyroptosis in Activated Macrophages LPS-activated BMDMs were infected with Y. pestis Δ1234 (T3SS^+^, type III effector^−^) or pCD1^−^ (T3SS^−^). (A) Macrophages were stained with FAM-YVAD (green) to identify cells with active caspase-1, Alexa633-phalloidin to visualize actin (blue), and anti-*Yersinia* antibodies (red) 90 min postinfection and examined by confocal microscopy. Representative of two experiments. (B) Membrane breakdown and LDH release from infected macrophages was inhibited by YVAD. Data shown are from three replicates and representative of two experiments. * *p* < 0.0001.

*Yptb* is closely related to Y. pestis, the causative agent of plague, and Yersinia spp. share several features including the plasmid encoded type III secretion apparatus [2]; therefore, we hypothesized that Y. pestis infection of activated macrophages would also result in pyroptosis. Activated macrophages were infected with a Y. pestis mutant competent for type III translocation, but lacking all translocated effectors (Δ1234) [55]*.* Like *Yptb*, Y. pestis causes activation of caspase-1 as demonstrated by staining with FAM-YVAD (Figure 8A, top). Y. pestis lacking the T3SS-encoding virulence plasmid (pCD1^−^) failed to induce FAM-YVAD staining in activated macrophages (Figure 8A, bottom), suggesting caspase-1 activation induced by Y. pestis also requires the T3SS. Infection of activated macrophages with Y. pestis also resulted in LDH release that was blocked by the caspase-1 inhibitor YVAD (Figure 8B); indicating Y. pestis contains the ligand responsible for caspase-1 activation, and this leads to caspase-1-dependent lysis of activated macrophages.

## Discussion

Our results demonstrate the ability of macrophage activation to fundamentally alter the host response to *Yptb* infection. In naïve macrophages, the YopJ-mediated inhibition of proinflammatory signaling [13–15] and induction of apoptosis [3,4,35] have been well described. However, in activated macrophages YopJ no longer functions in this capacity, and activation of the apoptotic executioner, caspase-3, is suppressed such that activation of macrophages results in susceptibility to caspase-1-dependent pyroptosis. The features of pyroptosis in *Yptb-*infected macrophages included early membrane permeabilization followed by DNA damage, and inflammatory cytokine processing and release. Macrophage activation may enhance sensitivity to pyroptosis by increasing synthesis of host proteins involved in triggering the activation of caspase-1 in response to *Yersinia*; pyroptosis is not observed during infection of naïve macrophages with YopJ^−^ *Yptb*. Alternatively, macrophage activation may overcome the ability of translocated *Yersinia* effector proteins to inhibit the activation of caspase-1 [56]. This is the first report of proinflammatory pyroptosis induced by wild-type *Yersinia*, bacteria previously thought to neutralize macrophages exclusively by noninflammatory apoptosis.

In vitro, *Yersinia* species are capable of suppressing inflammatory cytokine production in response to bacterial products [13–15], and during the early phase of infection in vivo, there is a marked lack of inflammation and inflammatory cytokine production [23,24]. This suggests that the majority of macrophages interacting with *Yersinia* in vivo would have a naïve phenotype, and this is consistent with the YopJ-dependent macrophage death observed [19,21], and the lack of caspase-1 activation when the bacterial burden is low and macrophages are not activated (Figure S4; Table S1). However, as infection progresses, histological examination of *Yersinia*-infected tissues reveals extensive inflammation and inflammatory cytokine production [24–28]. We have demonstrated that the inflammatory nature of *Yptb* infection leads to macrophage activation and up-regulation of surface ICAM-1, indicating that macrophages become resistant to YopJ and sensitive to pyroptosis. This result was confirmed by the finding that activated macrophages from *Yptb-*infected mice also contain active caspase-1, and this process required the T3SS-encoding virulence plasmid, and therefore was likely dependent on the T3SS. In vivo, we have not formally excluded the involvement of plasmid-encoded gene products that are not part of the T3SS in caspase-1 activation; however, we feel this is unlikely considering our results confirming the T3SS-dependence of pyroptosis in vitro. T3SS-dependent pyroptosis and inflammatory cytokine production may help explain the ability of T3SS+ *Yptb* to induce greater levels of tissue necrosis than T3SS^−^ *Yptb* [28], even in the presence of type III effectors capable of suppressing inflammation [2]. Our results suggest that during *Yersinia* infection in vivo macrophages encounter TLR or other activating ligands that trigger a host-mediated switch from YopJ-dependent apoptosis to pyroptosis. In addition, we predict macrophage populations that are continuously encountering bacterial products, like those in the Peyer's patches (PPs), would be refractory to the effects of YopJ. Consistent with this hypothesis, YopJ does not confer a replicative advantage in the PPs; YopJ^−^ *Yptb* replicate as well as wild-type in the PPs of infected mice [19,21]. PP macrophages may also be inherently susceptible to pyroptosis; unfortunately, we were unable to analyze caspase-1 activation in the PPs due to the low numbers of cells present.

Superficially, *Yersinia* infection of activated or naïve macrophages simply results in host cell death; however, the responses of other host cells to apoptosis and pyroptosis are quite different. Apoptotic cells often display surface markers that facilitate their uptake by neighboring cells [57] and prevent release of inflammatory intracellular contents from dying cells. Phagocytes that encounter apoptotic cells produce anti-inflammatory cytokines TGF-β and IL-10 and produce lower levels of several inflammatory cytokines [58,59] and costimulatory molecules [60]. This potent anti-inflammatory response is able to modulate the adaptive immune response by reducing the ability of antigen-presenting cells to stimulate T cells [61]. The anti-inflammatory nature of apoptosis is consistent with the ability of YopP (functionally equivalent to YopJ) to delay priming of T cells during Y. entercolitica infection [62].

In contrast, pyroptosis is intrinsically inflammatory, as the cell death process is linked to maturation and release of inflammatory cytokines. Pyroptosis also results in rapid lysis and release of intracellular contents [38,41] that can act as “danger signals” and promote the immune response [61,63]. *Yersinia-*induced macrophage death could result in drastically different outcomes depending on the activation state of the macrophage, even though the immediate consequence in both naïve and activated macrophages is simply cell death. The role of IL-18 and IL-1β in enhancing immune responsiveness has been thoroughly demonstrated. Both induce inflammatory cytokine production and increased expression of adhesion molecules, recruiting neutrophils and lymphocytes to sites of infection [64]. Correspondingly, *Yersinia-*infected mice have increased numbers of neutrophils in colonized tissues [26,28]. IL-18 also plays a major role, in conjunction with IL-12, in stimulating interferon gamma production [43]. Depending on the cytokine milieu, IL-18 can stimulate CD4+ T cell differentiation to Th1 or Th2 phenotype [65]. Both IL-18 and T cell responses are critical in controlling *Yersinia* infection in vivo, as IL-18–deficient mice [66] and mice lacking T cells [67] are unable to resolve the infection, and adoptive transfer of *Yersinia*-specific T cells confers partial protection against challenge [68]. Thus, redirecting macrophages to undergo pyroptosis appears to play an important role in generating an appropriate and effective immune response to *Yersinia*.

The activation of caspase-1 is initiated by recognition of cytosolic ligands by members of the NOD-leucine-rich repeat family of proteins. This recognition triggers formation of a multiprotein complex called the inflammasome, which then acts as a platform for the activation of caspase-1 [69]. Induction of pyroptosis by *Yptb* requires the bacterial T3SS but none of its known effectors. We hypothesized that the *Yersinia* T3SS actively or passively transports a caspase-1–activating ligand into the macrophage cytosol. Recent studies with *Salmonella* and *Legionella* have implicated cytosolic flagellin in activating caspase-1 through the NOD-like receptor family member Ipaf [70–73]. Y. pestis strains have a mutation inactivating *flhD* [74] that results in suppression of flagellin subunit production, and the observed lack of motility and flagella [75]. Our observation that Y. pestis also induces caspase-1 activation suggests the delivery of an alternative caspase-1 activating ligand to the host cytosol, and experiments to identify the ligand(s) produced by *Yersinia* species are ongoing.

Bacterial pathogens are often capable of modulating host cell processes, including cell death. Pathogens prevent cell death to maintain a protective intracellular environment or replicative niche [76,77], or induce cell death to eliminate host cells and suppress immune function [5,78]. In addition, activation of caspase-1-dependent inflammatory programmed cell death, or pyroptosis, in response to cytosolic bacterial ligands may serve as a host defense mechanism [70–73]. This study demonstrates host-mediated redirection of *Yersinia-*induced cell death; recognition of host inflammatory mediators and bacterial products results in inhibition of apoptosis, a noninflammatory process thought to benefit the bacteria, and primes macrophages to die by pyroptosis, potentially benefiting the host by shifting host cell responses toward inflammation.

## Materials and Methods

### Bacterial strains and growth conditions.

*Yptb* strains used in the present study were wild-type (YPIII) and the following mutants derived from this strain: YP26 YopJ^−^ [15], YP18 YopB^−^ [15], and YP37 YopJOEHKM^−^ [79] (a gift from Dr. James Bliska). A plasmid expressing green fluorescent protein was generated by inserting the LacZ promoter (bases 246–575) from pBluescriptSK− into the EcoRI and BamHI sites of pDW1 [80]. A yopE::cyaA fusion was constructed as described [36] and inserted into the HindIII and BamHI sites of pBR322. pIB1^−^ *Yptb* were generated as described [81] and screened by PCR to confirm loss of multiple *yop* genes. Bacteria were routinely cultured in LB at room temperature.

For macrophage infections, overnight cultures were back-diluted 1:40 into LB containing 20 mM sodium oxalate and 20 mM magnesium chloride and grown at room temperature with shaking for 1 h followed by incubation at 37 °C with shaking for 2 h. Bacteria were harvested and resuspended in PBS for infection.

Yersinia pestis strains used in the present study were KIM8 Δ1234 [55] and pCD1^−^ plasmid-cured (a gift from Dr. Greg Plano). Y. pestis was grown as described for *Yptb* and sonicated briefly prior to infection to reduce clumping.

Heat-killed YPIII were prepared by growing the bacteria as for infection, washing cells and resuspending in PBS, and incubating at 65 °C for 1 h.

### Macrophages and infection.

Bone marrow-derived macrophages (BMDMs) were isolated from the femur exudates of C57BL/6 mice (Jackson Laboratories) and cultured at 37 °C in 5% CO_2_ in Dulbecco's minimal essential medium (DMEM, Invitrogen) supplemented with 10% FCS, 5 mM HEPES, 0.2 mg/ml L-glutamine, 0.05 mM β-mercaptoethanol, 50 mg/ml gentamicin sulfate, and 10,000 U/ml penicillin and streptomycin with 30% L-cell-conditioned medium [82]. After 6–7 d of incubation, macrophages were collected by washing with ice-cold PBS containing 1 mM EDTA, resuspended in supplemented antibiotic-free DMEM containing 5% FCS, and allowed to adhere for 18–24 h before infection. Macrophages were activated with ultrapure LPS from Salmonella minnesota (List Biologicals) at a final concentration of 100 ng/ml unless otherwise indicated, 100 ng/ml Pam_3_CSK (EMC microcollections), or heat-killed wild-type YPIII for 18 h prior to infection. Medium was replaced 1 h before infection, and contained 200 μM YVAD.cmk and zFA.fmk (Calbiochem) when indicated. Bacteria were added at a multiplicity of infection of 20 and spun briefly at 200 *g* to bring bacteria into contact with macrophages. Gentamicin sulfate was added to 100 μg/ml at 2 h.

Efficiency of infection was confirmed by infection with GFP-expressing *Yptb* followed by incubation for 5 min or 2 h. Macrophages were stained using Texas Red-phalloidin or Alexa 633-phalloidin per the manufacturers instructions and examined by confocal microscopy using the BioRad MRC-600 or Leica SL confocal microscope in the W. M. Keck Center for Advanced Studies in Neural Signaling (University of Washington, Seattle, WA). The number of macrophages with associated bacteria was determined from multiple fields.

### Lactate dehydrogenase release.

Macrophages grown in 96-well plates were infected with *Yptb*, and supernatants were evaluated for the presence of the cytoplasmic enzyme LDH using the Cytotox 96 kit (Promega) as directed by the manufacturer's instructions. Percentage cytotoxicity was calculated as 100 × (experimental LDH − spontaneous LDH) ÷ (maximum LDH release − spontaneous LDH).

### CyaA-based translocation assay.

Macrophages were infected with *Yptb* containing pYopE::cyaA at an MOI of 20 for 1–2 h. 2 × 10^4^ macrophages were lysed in 0.1 M HCl, and cAMP levels were determined by using the Direct cAMP Correlate-EIA Kit (Assay Designs) and normalized for protein content determined by the Bradford Protein Assay (Bio-Rad).

### Caspase-3 activity.

Macrophages were infected with *Yptb* for the indicated length of time and the Caspase-3/7 Glo Assay (Promega) was performed according to the manufacturers instructions. Briefly, 60 μl of Caspase-3/7 Glo reagent was added to 1 × 10^4^ macrophages in 60 μl of medium and incubated at room temperature for 1 h. Luminescence was measured using a TECAN GENios Pro. Caspase-3/7 activity is reported as relative light units (RLU) of infected samples minus uninfected control.

For ICAD immunobloting, 1.5 × 10^6^ macrophages were harvested at 2 h postinfection and lysed in sample buffer. Proteins were separated by 15% SDS-PAGE and transferred to nitrocellulose membranes. ICAD cleavage was assessed by Western blotting using anti-ICAD antibodies and peroxidase conjugated secondary antibodies (BDPharMingen). Immunoblots were developed with and enhanced chemiluminescence system (Amersham Biosciences). Anti-p44/p42 antibodies were used to confirm equal loading.

### Ethidium bromide/SYTO10 staining.

Macrophages grown on glass coverslips were infected with *Yptb* for the indicated length of times. Media was removed and adherent cells were stained with SYTO 10 (Molecular Probes) and ethidium bromide at 25 μg/ml (Sigma-Aldrich) in HBSS for 5 min. Coverslips were analyzed using a BioRad MRC-600 or Leica SL confocal microscope in the W. M. Keck Center for Advanced Studies in Neural Signaling. The means and standard deviations (SDs) were derived from counting three fields for uninfected samples and six fields for infected samples.

### DNA fragmentation assays.

Macrophages grown on glass coverslips were infected with *Yptb*, and DNA strand breaks were detected using terminal deoxynucleotidyl transferase-mediated dUTP nick end-labeling (TUNEL) using the In Situ Cell Death Detection Kit as directed by the manufacturer's instructions (Roche Applied Science). Coverslips were mounted using ProLong antifade (Molecular Probes) and analyzed using a BioRad MRC-600 or Leica SL confocal microscope in the W. M. Keck Center for Advanced Studies in Neural Signaling. The means and SDs were derived from counting three fields for uninfected samples and six fields for infected samples.

### Caspase-1 staining in vitro.

Macrophages grown on glass coverslips were infected with *Yptb* for 90 min total and carboxyfluorescein-YVAD-fluoromethyl ketone (FAM-YVAD; Immunochemistry Technologies) was added to 1× at 30 min postinfection. Macrophages were washed thoroughly to remove unbound FAM-YVAD and then stained with Alexa 633 phalloidin (Molecular Probes) per the manufacturers instructions. Bacteria were labeled using anti-*Yptb* and anti-rabbit PE (Abcam) antibodies. Coverslips were mounted using ProLong antifade (Molecular Probes) and analyzed using Leica SL confocal microscope in the W. M. Keck Center for Advanced Studies in Neural Signaling.

### IL-18 secretion.

Macrophages were infected in serum-free media, and at the indicated time points the supernatant was removed, sterilized using a 0.22 μm filter, and concentrated using a 10,000 MWCO Centricon Plus-20 centrifugal filter device (Millipore). Supernatant from 2.4 × 10^6^ macrophages was separated by 15% SDS-PAGE, transferred to nitrocellulose membranes, and cytokine processing and release was analyzed by western blot using anti-IL-18 M19 and peroxidase-conjugated secondary antibodies (Santa Cruz Biotechnology). Immunoblots were developed with an enhanced chemiluminescence system (Amersham Biosciences).

### Mouse infections.

Female C57BL/6 mice aged 6–8 wk (Jackson Laboratories) were infected orogastrically with 6−8 × 10^8^ wild-type or pIB1^−^ *Yptb* in 100μl of PBS. Mice were sacrificed on days 4, 5, and 6 postinfection, and spleen and MLNs were removed and placed in cold PBS. Organs were homogenized between frosted glass slides. An aliquot was removed and lysed in 1% triton for CFU determination by plating dilutions on cefulosodin-irgasan-novobiocin (CIN) agar. The remaining cells were processed for staining: red blood cells were lysed in 17 μM Tris (pH 7.4), 140 μM NH_4_Cl for 5 min at room temperature, washed once in cold PBS, and passed through a 70 μM filter to create a single cell suspension. Cell numbers were determined by Trypan blue exclusion. To identify activated macrophages, 2 × 10^6^ cells were stained with anti-F4/80-PE antibodies (Caltag), anti-ICAM1-biotin antibodies, and streptavidin-APC (BD Pharmingen) on ice for 30 min. Cells were fixed and analyzed by flow cytometry using a BD LSR 6 color analyzer. Isotype control antibodies resulted in an MFI equivalent to that of unstained cells. To identify macrophages with active caspase-1, 2 × 10^6^ cells were incubated for 30 min at 37 °C with 5% CO_2_ with 1× FAM-YVAD in PBS supplemented with 5 mM glycine to reduce cell breakdown [41]. Cells were then washed thoroughly to remove unbound FAM-YVAD and labeled with anti-F4/80-PE antibodies, fixed, and analyzed by flow cytometry. Increased FAM-YVAD staining was not due to cross reactivity with caspases activated during *Yptb*-induced apoptosis; naïve macrophages infected with wild-type *Yptb* did not have increased FAM-YVAD staining.

Animal experiments were approved by the University of Washington Institutional Animal Care and Use Committee, Seattle, WA.

## Supporting Information

Figure S1 Wild-Type *Yptb* Induces Bona Fide YopJ-Dependent Apoptosis in Naïve Macrophages Naïve BMDMs were infected with wild-type or YopJ^−^ *Yptb* in the same experiment reported in Figure 2.
(A) Macrophages labeled with SYTO10 (green) were stained with membrane-impermeant EtBr (MW = 394 Da, red) and examined by confocal microscopy to assess increases in membrane permeability (EtBr-positive/SYTO10-labeled, yellow). Representative images are shown.
(B) The percentage of EtBr-positive/SYTO10-labeled cells was determined; data shown are means and SDs from multiple fields. Representative of two experiments.
(C) DNA damage was assessed by TUNEL and confocal microscopy. Representative images are shown.
(D) The percentage of TUNEL-positive cells was determined; data shown are means and SDs from multiple fields. Representative of two experiments. Increased DNA damage was observed at 60 min (D) *p = 0.0002, prior to any significant increase in membrane permeability (B) ^*p* = 0.1 (no significant difference), and these features were dependent on YopJ. This result differs from infection of activated macrophages, in which wild-type and YopJ^−^ *Yptb-*induced membrane permeability precedes DNA damage by 120 min (Figure 2C and 2D).
(E) YopJ-dependent apoptosis in naïve macrophages was accompanied by activation of apoptotic caspases-8 and −3 [9,16]. Data presented are fold changes in caspase activity in cell lysates of infected macrophages compared with uninfected controls at 120 min. Means and SDs were calculated from three replicates and are representative of two experiments.
(F) YopJ-dependent DNA fragmentation in naïve macrophages was dependent on the apoptotic executioner caspase-3. Naïve BMDMs were treated with the caspase-3 inhibitor DEVD (200 μM) and infected with wild-type *Yptb.* DNA damage was assessed by TUNEL and confocal microscopy. The percentage of TUNEL-positive cells was determined; data shown are means and SDs from multiple fields. Representative of two experiments. * *p* = 0.0001.
(G) TLR4KO macrophages have reduced susceptibility to YopJ-dependent apoptosis as previously described [6,7]. Naïve wild-type and TLR4KO C57BL/6 macrophages were infected with wild-type and YopJ^−^ *Yptb*, and LDH release was measured 4 h after infection. Data shown are means of LDH release from three replicates and are representative of two experiments. *p = 0.0007.
(2.1 MB TIF)

Figure S2 Characteristic Apoptotic Nuclear Condensation during Infection of Naïve Macrophages with Wild-Type *Yptb* Contrasts with the Uniform Nuclear Distribution of Damaged DNA in Activated Macrophages Infected with *Yptb*
We examined nuclear morphology of cells immediately after the appearance of damaged DNA (TUNEL positivity) during infection of naïve and activated macrophages with *Yptb*. During YopJ-dependent apoptosis, TUNEL-positive nuclei undergo nuclear condensation and fragmentation characteristic of apoptosis [3,4,35].
(A) Naïve macrophages were infected with wild-type *Yptb*; TUNEL-positive nuclei demonstrating typical apoptotic condensation indicated by open arrowheads.
(B) In contrast, activated macrophages infected with wild-type or YopJ^−^ *Yptb* became TUNEL positive without marked nuclear condensation, and damaged DNA remained evenly distributed within the nucleus, suggestive of the previously reported morphology associated with caspase-1-dependent cell death [38].
(973 KB TIF)

Figure S3 Time Course of Macrophage Activation in Mice Infected with Wild-Type *Yptb*
At 4, 5, and 6 d postinfection, mesenteric lymph nodes (A) and spleens (B) were harvested from mice infected with wild-type *Yptb* and CFUs were quantified. In parallel, macrophage activation was assessed by ICAM-1 expression on F4/80+ macrophages from infected tissues. A greater than 2-fold increase in the percentage of ICAM-1^hi^ macrophages relative to macrophages from uninfected mice was scored as activated (red circles, activated; black circles, not activated). Data in (A) and (B) are from two independent experiments. ND, none detected.
(C) ICAM-1 expression on F4/80+ macrophages from MLNs (top row) and spleen (bottom row) and corresponding colony-forming units (CFU) from one time point represented in (A) and (B).
(1.8 MB TIF)

Figure S4 Macrophage Activation and Caspase-1 Activation in Mice Infected with Wild-Type *Yptb*
Spleens were harvested from mice infected with wild-type *Yptb*, and macrophage activation was analyzed by ICAM-1 expression (top row with corresponding CFU). Caspase-1 activity (bottom row) was examined in parallel by FAM-YVAD staining. Red histograms were scored as *Yptb-*infected with activated macrophages and black histograms as *Yptb-*infected without activated macrophages. ND, none detected. Caspase-1^hi^ macrophages have higher levels of surface ICAM-1 than caspase-1^lo^ macrophages (see Figure 7E and 7F).
(1.5 MB TIF)

Table S1 Caspase-1 Activation in Mice Infected with Wild-Type *Yptb*
Percentage of active caspase-1^hi^ macrophages from uninfected and infected macrophage populations averaged from two independent experiments, including Figure S4.
(25 KB DOC)

### Accession Numbers

The GenBank (http://www.ncbi.nlm.nih.gov/Genbank/) GI numbers for the genes and gene products discussed in this paper are: *Yptb* YopJ (56405330); *Yptb* YopB (549791); caspase-1 (86198305); TLR4 (10946594); IL-18 (6680413); caspase-3 (118129865); ICAD (141802948); ICAM-1 (30172560).

## Abbreviations

BMDM: bone marrow-derived macrophage
CFU: colony-forming units
Cya: calmodulin-dependent adenylate cyclase
EtBr: ethidium bromide
IL: interleukin
GFP: green fluorescent protein
LDH: lactate dehydrogenase
LPS: lipopolysaccharide
MLN: mesenteric lymph node
MAPK: mitogen-activated protein kinase
NF-κB: nuclear factor kappa B
PP: Peyer's patch
SD: standard deviation
T3SS: type III secretion system
TLR: Toll-like receptor
TUNEL: terminal deoxynucleotidyl transferase-nick end labeling
*Yptb*: *Yersinia pseudotuberculosis*
