# Supplementary material for: Macrophage Activation Redirects Yersinia-Infected Host Cell Death from Apoptosis to Caspase-1-Dependent Pyroptosis
Source: PLoS Pathog. 2007 Nov 2;3(11):e161. doi: 10.1371/journal.ppat.0030161 (PMC2048529; doi:10.1371/journal.ppat.0030161)
Supplement: Supplementary file 1 [file ppat.0030161.st001.doc]

|  | % caspase-1^hi^ | *p*-value |
| --- | --- | --- |
| uninfected | 12.2% +/-1.86% |  |
| *Yptb-*infected w/o macrophage activation | 13.4% +3.87% | vs. uninfected = 0.58 |
| *Yptb-*infected w/macrophage activation | 29.0% +/-5.76% | vs. uninfected = 0.0009 vs. infected w/o act. = 0.0005 |
